# Effect of processing method (natural, washed, honey, fermentation, maceration) on the availability of heavy metals in specialty coffee

**DOI:** 10.1016/j.heliyon.2024.e25563

**Published:** 2024-02-01

**Authors:** Matúš Várady, Jana Boržíková, Peter Popelka

**Affiliations:** a Department of Food Hygiene, Technology and Safety, University of Veterinary Medicine and Pharmacy, Komenského 73, 041 81, Košice, Slovak Republic; b State Veterinary and Food Institute Dolný Kubín, Hlinkova 619, 043 65, Košice, Slovak Republic

**Keywords:** Anaerobic fermentation, Carbonic maceration, Coffee processing, Heavy metals, Specialty coffee

## Abstract

The aim of this study was to determine the effect of various methods of processing, such as natural, washed, honey, anaerobic fermentation, and carbonic maceration, on the contents of heavy metals in green and roasted specialty coffees from various countries of origin (Ethiopia, Kenya, Rwanda, Burundi, Guatemala, Nicaragua, and Peru). The heavy metals aluminium (Al), nickel (Ni), chromium (Cr), cadmium (Cd), copper (Cu), and lead (Pb) were identified by a multi-element technique using inductively coupled plasma mass spectrometry. Mercury (Hg) content was determined by atomic absorption spectrometry. The processing method affected the contents of Hg, Al, Ni, Cr, Cd, and Pb in the green and roasted coffees (*p* < 0.001). Hg content varied in the green coffees processed by fermentation methods *vs* natural or washed methods (i.e. Rwandan and Guatemalan coffees). Cd content was highest in Guatemalan green coffee processed using carbonic maceration (0.062 mg/kg). Pb content differed between the Ethiopian and Rwandan roasted coffees, with the highest content in the washed method (0.252 mg/kg). The correlations between the contents of Cu and Al, Ni and Cr, and Pb and Cr were significant for both the roasted and green beans. In conclusion, the method of processing can affect the contents of heavy metals in green and roasted specialty coffees. Monitoring heavy metals when processing coffee with new methods, even though further processing such as roasting can substantially reduce their content in some cases, is therefore important.

## 1 Introduction

Coffee is one of the most popular drinks in the world due to its sensory properties and the stimulating effect of caffeine [1]. Coffee contains many beneficial substances but can also contain potentially harmful substances due to improper processing, subsequent handling, or low-quality green beans [2,3]. Specialty coffee is the term used for coffee that has a standardised process of production, from choosing criteria for coffee plantations to coffee brews that pass preliminary tests of grading and cupping before being served to the client [4,5]. One of the most important criteria is to achieve a cupping score of 80 points or above on the 100-point scale [4]. Specialty coffees have the highest quality on the market, and their processing is one of the most important activities affecting the quality and safety of the beans. To maintain the high quality of specialty coffees, older processing methods (natural, washed, and honey) are gradually being replaced by new methods, e.g. anaerobic fermentation, carbonic maceration, hypoxia, and thermal shock [6,7]. Various methods of post-harvest processing can determine the sensory profile, producing coffees with highly differentiated characteristics, classified as specialty coffees [8].

Heavy metals are elements with atomic weights between 63.5 and 200.6 g/mol. They are extremely harmful to human health even in very low concentrations because they accumulate in the food chain [[9], [10], [11]]. Some metals, including copper (Cu), chromium (Cr), cobalt, nickel (Ni), and zinc, are biologically essential to organisms at low concentrations. Elements such as arsenic (As), cadmium (Cd), lead (Pb), mercury (Hg), titanium, and uranium, however, are not essential and are harmful. All metals are toxic at high concentrations, and excessive amounts can damage the body [12]. The oral route is a common means of ingesting heavy metals, and excessive levels can cause substantial damage to every organ in the body and other problems such as neurological defects, respiratory disorders, carcinogenicity, gastrointestinal obstruction, and osteoporosis [13]. Heavy metals are associated with some diseases, e.g. kidney failure, blood and lung cancers, skeletal deformities, and osteoporosis [14]. Studies that analyse the concentrations of toxic heavy metals in widely consumed foods are therefore beneficial to protect human health from the harmful effects of hazardous metals [15,16]. The bioavailability and occurrence of heavy metals, however, are influenced by other factors such as soil pH and texture, organic matter content, and interactions among elements [17]. Farmers only use sensorial analyses to evaluate the quality of specialty coffee. Specialty coffee is considered to have the highest quality on the market (it is only 5 % of the entire market), but the customer, roaster, and farmer do not know if it also contains heavy metals. The contents of heavy metals in green and roasted coffees processed using the new methods have rarely been reported [18]. The concentrations of heavy metals in commercially roasted ground coffee are lower than the limits recommended by the official inspection agencies and are suitable for consumption [19,20]. The new methods of processing coffee are mainly aimed at optimising the fermentation of coffee cherries and beans, including the formation of volatiles, flavour precursors, organic acids, and bioactive compounds [7].

A recent study analyzed various coffee-processing methods (natural, washed, honey, anaerobic fermentation, and carbonic maceration) and their effects on bioactive and volatile compounds in different countries of origin or within the same farm [6]. These new innovative processing methods, especially anaerobic fermentation and carbonic maceration, however, rapidly alter the characteristics of the coffee fruit, leading to a taste very different from traditional fermentation methods. We thus hypothesised that the method of processing would also strongly affect the content of heavy metals in specialty coffee beans. The novelty of our study is the comparison of the contents of heavy metals in specialty coffee processed by different methods that have recently been used. Our objectives were therefore to determine: (1) the effect of the processing method on the contents of heavy metals (Al, Hg, Ni, Cr, Cd, Cu, and Pb) in green specialty coffee, (2) the effect of processing method on the contents of heavy metals (Al, Hg, Ni, Cr, Cd, Cu, and Pb) in roasted specialty coffee, and (3) whether different processing methods affected the heavy-metal contents in specialty coffees from the same farm.

## 2 Materials and methods

### 2.1 Coffee samples

Samples of green and roasted beans of 100 % *Coffea arabica* from Ethiopia (Gedeo), Ethiopia (Sidama) Kenya, Burundi, Rwanda, Guatemala, Nicaragua, and Peru were used. Heirloom Ethiopian beans (ETH) obtained from the Gedeo (Yirgacheffe) region were harvested in 2021 and processed using two methods, natural and washed as was previously described [6]. Kenyan (KEN) beans were obtained from the Meru region. The beans were isolated from Ruiru 11 variety harvested in 2021 and were processed using two methods, natural hypoxia and washed. Hypoxia is a state in which oxygen is not available in sufficient amounts, i.e. not the complete absence of oxygen, compared to anaerobic fermentation [21]. Rwandan (RWA) beans were obtained from the Nyamasheke district, Western Province. The beans were the Red Bourbon variety harvested in 2021 and processed naturally or using anaerobic fermentation. Beans processed using anaerobic fermentation were transferred to a tank where they were anaerobically fermented for 60 or 100 h, after which they were dried on raised African beds for about 25–30 d. Guatemalan (GUA) beans were obtained from Finca La Senda in the Chimaltenango region of Acatenango. The beans were the Catuai variety harvested in 2021 and processed using four methods, washed, carbonic maceration (GUA-CM), anaerobic fermentation-honey (GUA-AFH), and anaerobic fermentation for 250 h (AF). Cherries processed by carbonic maceration were placed into airtight plastic barrels and macerated (fermented) in an environment rich in CO_2_. The anaerobic fermentation (GUA-AFH) methods started with the natural dry method and then fermented for 250 h in closed tanks. The origin and processing of the NIC, BUR, PER, and ETH (Sidama) beans have already been described in detail [6].

### 2.2 Coffee roasting

Green beans were roasted at a final temperature of 204 °C for 5 min. The development time was about 30 s (the time after the first crack) [22], and the ratio between the development time and the total roasting time for each sample was 11–14 %. The beans were roasted in batches of 50 g in an Ikawa Pro100 sample roaster (Ikawa Ltd., London, United Kingdom).

### 2.3 Analysis of heavy metals

The coffee samples were ground to a standard grind, homogenised, weighed (0.3 g), and transferred to Merck Teflon beakers (Merck KGaA, Darmstadt, Germany). Five millilitres of 65 % nitric acid and 1 mL of hydrogen peroxide were added. The beakers were sealed, and the samples were mineralised for 45 min, as previously described [18]. The sample solutions were then quantitatively transferred to 25-mL volumetric flasks, and deionised water was added to bring the volume to 25 mL for analysis. The samples were digested in a Berghof MWS-2 microwave device (Berghof Automation GmbH, Eningen unter Achalm, Germany) for 45 min at temperatures ranging from 100 to 190 °C. Heavy metals (Al, Ni, Cr, Cd, Cu and Pb) were identified using the multi-element technique using an ICP-MS 7900 inductively coupled plasma mass spectrometer (Agilent Technologies, Inc., Santa Clara, USA). The ICP-MS conditions were: RF power, 1370 W; RF matching, 1.58 V; plasma gas (Ar) flow, 15 L/min; sampling depth, 7.9 mm; carrier gas (Ar) flow, 1.22 L/min; nebuliser pump, 0.1 rps; and spray-chamber temperature, 2 °C. Hg content was determined by atomic absorption spectrometry using an AMA-254 single-purpose atomic absorption spectrometer (Altec, Praha, Czech Republic).

### 2.4 Statistical analysis

Data were analyzed using GraphPad Prism 9.2.0 (332) 2021 (GraphPad Software, Inc., San Diego, CA, USA) using one-way analyses of variance. Individual differences were determined using Tukey's multiple-comparison post hoc test when the overall effect of processing was significant (*p* < 0.05). A correlation analysis (Pearson) was performed between heavy-metal contents in the green and roasted samples.

## 3 Results

### 3.1 Heavy-metal content in green coffee

The processing method often affected the contents of Hg, Al, Ni, Cr, Cd, Pb (*p* < 0.001), and Cu (*p* < 0.032) in the green specialty coffees from the same farm (Table 1). Hg content was highest in GUA processed using carbonic maceration (0.015 mg/kg) and differed from coffees processed using the washed, AFH, and 250-h fermentation methods. Hg content differed between the green coffees processed by fermentation methods *vs* natural or washed methods (RWA and GUA). Al, Ni, and Cr contents were highest in the ETH-Gedeo washed and KEN natural coffees. Cd content was highest in GUA green coffee processed using carbonic maceration (0.062 mg/kg), which differed from other GUA green coffees. Cu contents were similar in all green coffees, except for the low content in KEN green coffee processed using natural hypoxia. Pb content varied in the ETH and PER coffees and was highest in ETH coffees processed using the washed and fermentation (ACR) methods.

**Table 1:** Effect of processing methods on the content of heavy metals in green specialty coffee (mean ± SD, n = 3).

Coffee	Processing	Heavy metals (mg/kg)						
		Hg	Al	Ni	Cr	Cd	Cu	Pb
ETH- Gedeo	Washed	0.002 ± 0.002	15.52 ± 3.421^b^	0.717 ± 0.041^b^	0.454 ± 0.032^b^	0.007 ± 0.001^b^	6.801 ± 1.326	0.205 ± 0.032^b^
	Natural	0.002 ± 0.003	0.260 ± 0.302^a^	0.530 ± 0.017^a^	0.083 ± 0.001^a^	Nd^a^	11.03 ± 2.331	0.034 ± 0.002^a^
ETH-Sidama	EAO	0.001 ± 0.002	1.160 ± 0.061^b^	0.500 ± 0.053	0.095 ± 0.013^a^	0.006 ± 0.002^a^	6.601 ± 1.173	0.066 ± 0.011^a^
	ACR	0.001 ± 0.002	0.108 ± 0.002^a^	0.532 ± 0.022	0.216 ± 0.052^b^	0.014 ± 0.001^b^	10.48 ± 2.142	0.198 ± 0.030^b^
KEN	Washed	0.003 ± 0.001^b^	0.103 ± 0.712^a^	0.351 ± 0.027^a^	0.167 ± 0.002^a^	0.011 ± 0.001^b^	11.90 ± 2.221^b^	0.087 ± 0.031
	Natural	0.002 ± 0.002^a^	15.28 ± 1.761^b^	0.968 ± 0.043^b^	0.360 ± 0.003^b^	Nd^a^	2.940 ± 0.962^a^	0.080 ± 0.012
BUR	Natural	0.001 ± 0.003	0.182 ± 0.032	0.414 ± 0.031	0.149 ± 0.022	0.006 ± 0.001	10.14 ± 2.002	0.061 ± 0.022
	Anaerobic	0.001 ± 0.003	0.176 ± 0.041	0.483 ± 0.010	0.218 ± 0.014	0.005 ± 0.002	10.14 ± 1.923	0.050 ± 0.003
RWA	Natural	0.002 ± 0.001^a^	0.787 ± 0.084	0.792 ± 0.044	0.060 ± 0.002	0.005 ± 0.001	10.80 ± 2.402	0.019 ± 0.001
	60 h	0.003 ± 0.002^b^	1.490 ± 0.038	0.759 ± 0.063	0.076 ± 0.001	0.004 ± 0.001	10.90 ± 2.275	0.026 ± 0.003
	100 h	0.009 ± 0.001^c^	0.509 ± 0.054	0.764 ± 0.023	0.122 ± 0.002	0.004 ± 0.001	9.990 ± 1.807	0.040 ± 0.001
GUA	Washed	0.007 ± 0.003^a^	0.039 ± 0.001	0.226 ± 0.011	0.103 ± 0.004	0.004 ± 0.001^b^	11.29 ± 3.102	0.039 ± 0.001
	AFH	0.010 ± 0.003^c^	0.200 ± 0.004	0.111 ± 0.006	0.060 ± 0.003	0.005 ± 0.001^b^	11.20 ± 2.789	0.026 ± 0.001
	CM	0.015 ± 0.001^d^	0.203 ± 0.072	0.172 ± 0.052	0.097 ± 0.004	0.062 ± 0.004^c^	11.20 ± 3.001	0.093 ± 0.002
	250 h	0.009 ± 0.002^b^	2.450 ± 0.053	0.135 ± 0.030	0.101 ± 0.007	Nd^a^	11.33 ± 3.145	0.033 ± 0.001
NIC	Washed	0.001 ± 0.002	1.600 ± 0.003	0.435 ± 0.011^a^	0.122 ± 0.010^a^	0.022 ± 0.003^a^	10.30 ± 3.752	0.052 ± 0.003
	Honey	0.001 ± 0.002	0.621 ± 0.022	0.660 ± 0.063^b^	0.279 ± 0.022^b^	0.029 ± 0.003^b^	10.20 ± 2.077	0.091 ± 0.002
PER	Natural	0.001 ± 0.001	0.381 ± 0.007	0.144 ± 0.017^a^	0.106 ± 0.022^a^	0.005 ± 0.001^a^	10.97 ± 2.606	0.046 ± 0.001^a^
	Anaerobic	0.001 ± 0.001	1.070 ± 0.012	0.460 ± 0.012^b^	0.237 ± 0.081^b^	0.010 ± 0.002^b^	10.01 ± 2.200	0.085 ± 0.002^b^
*p*		0.001	0.001	0.001	0.001	0.001	0.032	0.001

SD – standard deviation; ETH – Ethiopian; KEN – Kenyan; RWA – Rwandan; BUR – Burundi; GUA – Guatemalan; NIC – Nicaraguan; PER, Peruvian; EAO – fermentation method (beans dried on African beds); ACR – fermentation method (beans dried under sleeping bags in the mountains on African beds); CM – carbonic maceration; AFH – anaerobic fermentation-honey; Hg – mercury; Al – aluminium; Ni – nickel; Cr – chromium; Cd – cadmium; Cu – copper; Pb – lead; Nd – not detected. Different letters within a column indicate significant differences between coffees from the same farm at *p* < 0.05.

### 3.2 Heavy-metal content in roasted coffee

The processing method often affected the contents of Hg, Al, Ni, Cr, Cd, and Pb in the roasted specialty coffees from the same farm (*p* < 0.001) (Table 2). Hg content in the GUA coffee was highest with the CM method (0.013 mg/kg). Al contents for the RWA and GUA roasted coffees were highest with the fermentation methods (i.e. 60-h fermentation and AFH, at 34.60 and 42.10 mg/kg, respectively). Ni content was highest in roasted RWA fermented for 60 h. Roasting had an inconsistent effect of processing on Cr content, except for the NIC coffees. The Cd content in the roasted coffees differed with the processing method, except for GUA and PER. Cu content did not differ between the roasted coffees (*p* = 0.480). Pb content differed in the ETH and RWA coffees and was highest in ETH-Gedeo processed using the washed method (0.252 mg/kg).

**Table 2:** Effect of processing methods on the content of heavy metals in roasted specialty coffee (mean ± SD, n = 3).

Coffee	Processing	Heavy metals (mg/kg)						
		Hg	Al	Ni	Cr	Cd	Cu	Pb
ETH- Gedeo	Washed	0.001 ± 0.001	9.201 ± 1.32	0.494 ± 0.023	0.075 ± 0.046	0.012 ± 0.002^a^	7.462 ± 1.437	0.252 ± 0.035^b^
	Natural	0.001 ± 0.001	0.710 ± 0.50	0.543 ± 0.017	0.091 ± 0.032	0.005 ± 0.001^b^	10.98 ± 2.440	0.057 ± 0.006^a^
ETH-Sidama	EAO	0.001 ± 0.001	4.310 ± 0.030	0.415 ± 0.042^a^	0.056 ± 0.040	Nd^a^	8.330 ± 2.421	0.038 ± 0.003^a^
	ACR	0.001 ± 0.001	19.83 ± 3.231	1.176 ± 0.050^b^	0.291 ± 0.041	0.017 ± 0.002^b^	11.77 ± 2.634	0.216 ± 0.045^b^
KEN	Washed	0.001 ± 0.001	29.39 ± 9.262	1.160 ± 0.073^b^	0.199 ± 0.044	0.008 ± 0.001^b^	12.25 ± 2.437	0.118 ± 0.062
	Natural	0.001 ± 0.001	13.49 ± 8.33	0.571 ± 0.032^a^	0.327 ± 0.035	0.005 ± 0.001^a^	9.791 ± 0.441	0.119 ± 0.075
BUR	Natural	0.001 ± 0.001	12.39 ± 0.075	0.303 ± 0.021	0.124 ± 0.022	0.005 ± 0.001^a^	11.27 ± 0.243	0.104 ± 0.012
	Anaerobic	0.001 ± 0.001	0.732 ± 0.056	0.353 ± 0.021	0.108 ± 0.011	0.007 ± 0.001^b^	11.27 ± 0.247	0.085 ± 0.020
RWA	Natural	0.002 ± 0.001^b^	0.031 ± 0.001^a^	0.615 ± 0.017^a^	0.064 ± 0.023	0.005 ± 0.001^a^	11.95 ± 1.997	0.033 ± 0.006^a^
	60 h	0.001 ± 0.001^a^	34.60 ± 5.943^c^	1.490 ± 0.022^c^	0.451 ± 0.012	0.009 ± 0.001^b^	11.02 ± 2.230	0.083 ± 0.006^ab^
	100 h	0.003 ± 0.001^c^	19.59 ± 1.942^b^	0.924 ± 0.063^b^	0.132 ± 0.045	0.005 ± 0.001^a^	10.59 ± 1.795	0.178 ± 0.046^b^
GUA	Washed	0.005 ± 0.002^b^	6.580 ± 0.005^a^	0.142 ± 0.032^a^	0.108 ± 0.046	Nd	11.76 ± 2.320	0.051 ± 0.002
	AFH	0.006 ± 0.003^c^	42.10 ± 17.42^b^	1.060 ± 0.022^b^	0.190 ± 0.071	Nd	12.10 ± 2.932	0.123 ± 0.053
	CM	0.013 ± 0.004^d^	16.35 ± 2.307^a^	0.437 ± 0.031^a^	0.129 ± 0.021	Nd	11.51 ± 2.123	0.063 ± 0.003
	250 h	0.002 ± 0.001^a^	3.600 ± 0.651^a^	0.208 ± 0.033^a^	0.104 ± 0.020	Nd	11.80 ± 2.442	0.049 ± 0.001
NIC	Washed	0.001 ± 0.001	0.597 ± 0.041	0.344 ± 0.021	0.162 ± 0.010^b^	0.020 ± 0.002^a^	11.96 ± 2.423	0.076 ± 0.003
	Honey	0.001 ± 0.001	0.238 ± 0.034	0.400 ± 0.011	0.115 ± 0.011^a^	0.029 ± 0.003^b^	10.64 ± 2.372	0.108 ± 0.061
PER	Natural	0.001 ± 0.001	2.770 ± 0.95	0.179 ± 0.046^a^	0.062 ± 0.021	0.006 ± 0.001	12.22 ± 2.441	0.065 ± 0.021
	Anaerobic	0.001 ± 0.001	18.24 ± 0.05	0.539 ± 0.052^b^	0.053 ± 0.031	0.006 ± 0.001	11.42 ± 2.024	0.180 ± 0.042
*p*		0.001	0.001	0.001	0.001	0.001	0.480	0.001

SD – standard deviation; ETH – Ethiopian; KEN – Kenyan; RWA – Rwandan; BUR – Burundi; GUA – Guatemalan; NIC – Nicaraguan; PER, Peruvian; EAO – fermentation method (beans dried on African beds); ACR – fermentation method (beans dried under sleeping bags in the mountains on African beds); CM – carbonic maceration; AFH – anaerobic fermentation-honey; Hg – mercury; Al – aluminium; Ni – nickel; Cr – chromium; Cd – cadmium; Cu – copper; Pb – lead; Nd – not detected. Different letters within a column indicate significant differences between coffees from the same farm at *p* < 0.05.

### 3.3 Correlation between heavy-metal contents in green and roasted specialty coffees

Pearson correlation coefficients were estimated for the contents of heavy metals in the green and roasted samples (Table 3). For the green coffees, Cu content was significantly correlated with Al, Ni, and Cr contents (*p* < 0.001, *p* < 0.01, *p* < 0.01, respectively), and Pb content was significantly correlated with Al and Cr contents (*p* < 0.05 and *p* < 0.001, respectively). For the roasted coffees, Ni content was significantly correlated with Al content, and Cr content was significantly correlated with Al and Ni contents (*p* < 0.001, *p* < 0.01 and *p* < 0.001, respectively). The correlations between the contents of Cu and Al, Ni and Cr, and Pb and Cr were significant for both roasted and green beans.

**Table 3:** Correlation matrices (i.e., Pearson's correlation coefficients) of analyzed heavy metals in green (G) and roasted (R) coffee.

	Hg G	Al G	Ni G	Cr G	Cd G	Cu G	Pb G	Hg R	Al R	Ni R	Cr R	Cd R	Cu R	Pb R
Hg G	1.000													
Al G	0.182	1.000												
Ni G	0.455	0.455	1.000											
Cr G	0.386	0.781	0.475^a^	1.000										
Cd G	0.430	0.188	0.2355	0.016	1.000									
Cu G	0.310	0.818^c^	0.594^b^	0.643^b^	0.204	1.000								
Pb G	0.218	0.460^a^	0.193	0.733^c^	0.259	0.322	1.000							
Hg R	0.876^c^	0.195	0.470^a^	0.301	0.708^c^	0.261	0.052	1.000						
Al R	0.358	0.052	0.082	0.113	0.002	0.139	0.044	0.222	1.000					
Ni R	0.050	0.073	0.304	0.090	0.0901	0.127	0.122	0.085	0.806^c^	1.000				
Cr R	0.011	0.177	0.341	0.046	0.058	0.142	0.064	0.111	0.603^b^	0.719^c^	1.000			
Cd R	0.508^a^	0.061	0.362	0.436	0.237	0.003	0.421	0.394	0.199	0.107	0.169	1.000		
Cu R	0.209	0.677^b^	0.514^a^	0.593^b^	0.118	0.754^c^	0.450	0.191	0.167	0.096	0.094	0.094	1.000	
Pb R	0.136	0.428	0.312	0.689^b^	0.044	0.253	0.760^c^	0.172	0.371	0.375	0.157	0.157	0.347	1.000

Hg – mercury; Al – aluminium; Ni – nickel; Cr – chromium; Cd – cadmium; Cu – copper; Pb – lead. ^a^*p* < 0.05, ^b^*p* < 0.01, ^c^*p* < 0.001.

## 4 Discussion

We compared samples of specialty coffees from the same country of origin and farm and of the same variety, but the samples differed by the way the coffee was processed. We analyzed the heavy-metal contents of the beans processed using the four main methods (natural, washed, honey, and fermented), with various methods of fermentation (anaerobic fermentation, carbonic maceration, AFH, EAO, ACR, and fermentation for 60, 100, and 250 h). A recent pilot study compared processing methods such as natural, washed, honey, anaerobic fermentation, and carbonic maceration and found differences in the contents of bioactive and volatile substances [6]. We were thus not surprised that the processing method in this experiment also affected the contents of heavy metals in the specialty coffees, even coffee from the same farm. Technological processes can generally affect the contents of heavy metals in commercially available roasted coffee [6,[23], [24], [25]]. The contents of some heavy metals in green beans changed after roasting but were still within the recommended limits [26].

The Hg content in our experiment was slightly higher in the green specialty coffees than the green coffees from Nicaragua and Colombia in our previous study, where the Hg content was 0.0007 mg/kg [18]. Kowalska [27], however, reported a higher Hg content of 0.006 mg/kg in Arabica beans. The fermentation methods in our experiment affected the Hg content the most in anaerobically fermented green and roasted coffees from Guatemala and Rwanda. Hg-resistant bacteria in surface water have recently been a major problem [28], so the possibility of a longer fermentation in water, which microorganisms can affect during processing, probably increases the Hg content. The fermentation of hulled beans can also increase the bioavailability of some elements, which can later be decreased by the new fermentation methods, especially by the activity of phytase released by microorganisms [29,30].

Al has become an indispensable material used in industry and modern daily life, so post-harvest processes can increase the content of this metal in beans regardless of the content in green coffee [31]. The methods of processing in our study affected the Al contents in the green coffee from ETH and KEN and in the roasted coffees from RWA and GUA. Different roasting technologies, and probably also storage and/or packaging, can further increase this content [32]. Al content was highest in the roasted RWA and GUA coffees, probably because some of the mass of the raw beans was lost during roasting, including water vapor, CO2, and other volatile compounds, so the content of some elements may increase proportionally during roasting [31]. A strong effect of the brewing technique on highly variable Al contents (1.5–15.5 mg/kg) in 10 samples of coffee beans has also been reported [32].

Ni content in our study was highest in the roasted KEN (washed), RWA (60-h fermentation), and ETH-Sidama (ACR) coffees (1.160, 1.490, and 1.176 mg/kg, respectively), slightly higher than in other studies [10,18]. Adler et al. [26] also reported higher average Ni contents in roasted (0.88 mg/kg) than green (0.42 mg/kg) commercially available coffee. De Carvalho Neto et al. [33] did not detect Ni in fermented roasted Brazilian coffee. The Ni contents of the green coffees in our study differed depending on the processing method, especially in ETH-Gedeo (washed *vs* natural), KEN (washed *vs* natural), NIC (washed *vs* honey), and PER (natural *vs* anaerobic fermentation), consistent with the Ni contents for the Columbian fermented and NIC natural green coffees [18]. Berego et al. [34], however, found much lower Ni contents (0.052–0.074 mg/kg) in washed and natural green coffees from Ethiopia. Ni content in our study, however, was significantly higher in roasted coffees, mainly in fermented coffee (ETH [ACR], RWA [60-h fermentation], and GUA [AFH]), but also in washed coffee (KEN).

The Cr and Cd contents in our study were generally low, but the absolute contents of elements in beans can vary due to different degrees of roasting, from light to dark [35]. Each coffee has a specific developmental period (time of development) [36], during which not only its sensory properties but also its bioactive and aromatic compounds are formed. Some coffees are generally suited to longer roasting times, so we assume that processing plays an important role in the contents of Cr, Cd, and Ni. Ni and Cr contents, however, are not currently legislated for coffee products. Cd content was highest in green GUA (CM), which differed from the other GUA green coffees, but Várady et al. [18] found an even higher Cd content (0.15 mg/kg) in Colombian coffee processed using anaerobic fermentation. Our Cd content of the GUA green coffee (0.062 mg/kg) was higher than in most other studies (e.g. 0.013, 0.006, and 0.03 mg/kg [[37], [38], [39]], respectively). In contrast, Kowalska [27] reported a high Cd content of 0.058 mg/kg in Arabica beans, which was comparable with the GUA green coffee in our experiment. We did not, however, detect Cd in the roasted GUA coffees. The maximum permitted level of Cd in coffee products set by the European Union is 100 μg/kg [10]. Albals et al. [39] found higher levels of Cd and Cr (0.450 and 1.540 mg/kg, respectively) in semi-roasted coffee, indicating that roasting can increase the contents of some elements.

Heavy-metal contents, though, can begin to decrease below the levels of green coffee during processing. These findings are consistent with our results for all coffees that were lightly roasted. Washed, natural, and fermented processing also influenced the Pb contents in the green ETH and PER coffees and the roasted ETH and RWA coffees. Colombian fermented and Nicaraguan natural green coffees in our previous study had slightly lower Pb contents (0.13 and 0.10 mg/kg, respectively [18]). One of the key heavy metals, Pb, is ubiquitous in the environment and can be easily absorbed by coffee beans from soil or water [25]. The Pb contents in the green coffees in our experiment varied with the processing method, e.g. ETH-Gedeo (washed *vs* natural), ETH-Sidama (EAO *vs* ACR), and PER (natural *vs* anaerobic fermentation). The Pb contents in the roasted coffees differed between ETH-Gedeo (washed *vs* natural), ETH-Sidama (EAO *vs* ACR), and RWA (natural *vs* fermented). Pb content was highest in the ETH-Gedeo coffee processed by the washed method (0.252 mg/kg). Da Silva et al. [10] reported higher Pb contents, with a maximum content of 1.5758 mg/kg in Brazilian roasted coffees, higher than the legally permissible range. Interestingly, Pb can be removed from roasted coffee by an activated-carbon filter system, which has been tested on samples with Pb contents above the permissible limits [40]. Van Cuong et al. [41] reported that Cu content gradually increased with the degree of roasting, with the highest content at 250 °C, but Pb content did not change after the roasting temperature increased. Jeszka-Skowron et al. [36] reported average Cu and Pb contents (13.3 and 0.023 mg/kg, respectively) in green Arabica beans. Cu content is probably slightly higher in Arabica than in Robusta beans. Berego et al. [34] did not detect Pb or Cd in samples of green coffee beans from Ethiopia, in contrast to our findings.

The correlations between Cu content and Al, Ni, and Cr contents and between Pb content and Cr content were significant for both the roasted and green beans. Roasting in some cases reduced the heavy-metal content of the specialty coffees from different countries of origin processed by different methods. It is possible that low or high levels of heavy metals are also due to differences in the origin of the coffee. The heavy-metal contents of specialty coffees, however, have rarely been studied, in contrast to commercial coffees. Specialty coffee, which has the highest quality on the market, should meet not only the highest sensory properties but also the highest properties of safety. Paying attention to new methods for processing coffee in the future is therefore very important.

## 5 Conclusion

The results of our study led to the conclusion that coffee processing could affect the contents of heavy metals in specialty coffees from the same farm. The contents of some heavy metals in green beans changed after roasting, depending on the processing method, but were still within the recommended limits. The contents of heavy metals in the green coffees differed between natural/washed and fermented coffees (anaerobic fermentation/carbonic maceration). Roasted fermented coffees, though, had higher contents of heavy metals than naturally fermented coffees. We therefore demonstrated the importance of monitoring heavy metals as part of entry controls when coffee processed using new methods enters the market, even though further processing such as roasting can in some cases strongly reduce their contents.

## Data availability statement

Data associated with the study has not been deposited into a publicly available repository. Data will be made available on request.

## CRediT authorship contribution statement

**Matúš Várady:** Writing – review & editing, Validation, Software, Methodology, Conceptualization. **Jana Boržíková:** Validation, Methodology. **Peter Popelka:** Supervision, Project administration.

## Declaration of competing interest

The authors Matúš Várady, Jana Boržíková and Peter Popelka of manuscript entitled: “**Effect of processing method (natural, washed, honey, fermentation, maceration) on the availability of heavy metals in specialty coffee**” declare that they have no known competing financial interests or personal relationships that could influence the results reported in this paper.
